# Prevention and treatment of a positive proximal margin after gastrectomy for cardia cancer

**DOI:** 10.1007/s13304-022-01315-4

**Published:** 2022-07-16

**Authors:** Eider Talavera-Urquijo, Andrew R. Davies, Bas P. L. Wijnhoven

**Affiliations:** 1 Department of Surgery, University Hospital of Donostia, Donostia-San Sebastián, Spain; 2 Department of Surgery, Guy’s and St Thomas’ NHS Foundation Trust, London, UK; 3 Department of Surgery, Erasmus University Medical Centre Rotterdam, Rotterdam, The Netherlands

**Keywords:** Positive resection margin, R1, Extended total gastrectomy, Cardia cancer, Junctional adenocarcinoma

## Abstract

A tumour-positive proximal margin (PPM) after extended gastrectomy for oesophagogastric junction (OGJ) adenocarcinoma is observed in approximately 2–20% of patients. Although a PPM is an unfavourable prognostic factor, the clinical relevance remains unclear as it may reflect poor tumour biology. This narrative review analyses the most relevant literature on PPM after gastrectomy for OGJ cancers. Awareness of the risk factors and possible measures that can be taken to reduce the risk of PPM are important. In patients with a PPM, surgical and non-surgical treatments are available but the effectiveness remains unclear.

## Introduction

The incidence of oesophagogastric junction (OGJ) adenocarcinoma has been gradually increasing both in Western and Asian countries [1, 2]. OGJ cancer involves subcardial, cardia and lower oesophageal adenocarcinomas. The Siewert classification is most often used to classify OGJ cancer. Three types are distinguished to facilitate interpretation of studies on OGJ cancer [3]. However, molecular subtyping of OGJ cancer has identified different subtypes which do not always correlate with the anatomical partition or patterns of nodal dissemination [4]. Hence, the optimal management of OGJ cancer remains uncertain [5].

Most surgeons would agree that the preferred surgical treatment for type I (lower oesophageal adenocarcinoma) is oesophagectomy with mediastinal lymph node dissection. For type III cancers (subcardial adenocarcinoma), total gastrectomy with partial oesophagectomy is performed [6]. For type II cancers (cardia cancer), both surgical approaches may be valid [7]. According to a survey extended to surgeons worldwide between 2013 and 2014, while Asian and South American centres seemed to perform extended total gastrectomy more often, European centres showed a more balanced proportion of the two approaches for Siewert II cancers [8]. Total gastrectomy facilitates a more complete abdominal (D2) lymphadenectomy, avoids postoperative morbidity related to a transthoracic approach and may result in less postoperative functional problems [9–11]. The downside of extended gastrectomy for type II cancer is the risk of an incomplete microscopic surgical resection (R1) at the proximal margin (oesophageal margin) [12]. On the other hand, oesophagectomy with gastric tube reconstruction via the transthoracic approach enables an extended two-field lymphadenectomy (abdominal and thoracic) and nearly eliminates the risk of a positive proximal margin. However, this approach is associated with a higher rate of respiratory complications and poorer functional outcomes [9, 10].

The main goal of any surgical treatment with curative intention is to achieve a complete resection of the tumour. However, the prevalence of a (microscopic) positive resection margin––proximal, distal or circumferential––after extended total gastrectomy for OGJ and gastric cancer ranges from 2.8 to 20% [13]. Here, in this narrative review, we discuss the risk factors, preventive strategies and (postoperative) treatments for positive proximal margin (PPM) after gastrectomy for OGJ cancer.

### Causes

A PPM is often only diagnosed on definitive histopathological examination of the resection specimen. This is generally due to an underestimation of the proximal extension of the tumour intraoperatively by the surgeon, often within the submucosal layer. It is therefore important to have a rigorous preoperative assessment by endoscopy and endoscopic ultrasonography (EUS). Three endoscopically visible landmarks should be assessed: the *Z*-line, gastric folds and the diaphragmatic pinch. Submucosal extension of the tumour makes it particularly difficult to judge the degree of oesophageal involvement. In this instance, EUS can be helpful. Furthermore, a minimum length of macroscopic oesophageal safety margin should be respected during surgical resection [14], although most guidelines do not report specifically on the required margin for OGJ cancer (Table 1).

**Table 1:** Guidelines on safe macroscopic resection margins for gastric and OGJ cancer

National guidelines	Indication
Japanese [15]	3 cm is recommended for T2 or deeper tumours with an expansive growth pattern and 5 cm for those with an infiltrative growth pattern. For T1 tumours, a gross resection margin of 2 cm should be obtained For tumours invading the esophagus, resection margin > 5 cm is not necessarily required, but frozen section examination of the resection line is preferable to ensure an R0 resection
ESMO-ESSO-ESTRO [16]	5 cm, if diffuse type 8 cm *Unspecific for OGJ cancer
NCCN [17]	4 cm or greater for T1b/T3. T4 tumours require en bloc resection of involved structures *Unspecific for OGJ cancer
AUGIS [18]	An ex vivo proximal margin of > 3.8 cm of normal oesophagus (which equates to 5 cm in vivo) The role of intraoperative histological examination of the proximal resection margin is mandatory in this situation
Dutch [19]	Proximal and distal margin of 6 cm intraoperatively When the distance to the proximal margin is less than 6 cm and a more extended resection would be possible if a frozen section would result positive, the use of frozen section analysis is recommended
German [20]	5 cm if intestinal Lauren’s pattern, 8 cm if diffuse type *Unspecific for OGJ cancer
French [21]	If R0 margins achievable, no distance required *Unspecific for OGJ cancer
Italian [22]	At least 3 cm is recommended for T2 or deeper tumours with an expansive growth pattern, and 5 cm is recommended for those with infiltrative growth pattern and diffuse Lauren histotype. For T1 tumours, margin of 2 cm For tumours invading the esophagus, a 5 cm margin is not necessarily required, but frozen section examination of the resection line is desirable to ensure an R0 resection

In some patients, the possibility of a PPM is accepted by the surgeon during the operation. For example, if the patient is considered too frail to undergo a more extensive resection. However, one should always consider the need to change the surgical approach and take this into account when selecting patients for total gastrectomy for OGJ cancer.

Distal gastrectomy may treat cancer-related symptoms (e.g., gastric outlet obstruction, bleeding) that are difficult to palliate with other modalities. In selected patients, this approach improves quality of life even when an incomplete tumour resection has been performed [23]. Total gastrectomy is associated with greater morbidity. Hence, many judge palliative total gastrectomy as too aggressive, also given the findings from the REGATTA trial that surgery does not prolong survival compared to chemotherapy alone [6, 24].

### Risk factors

Knowledge of the risk factors associated with a PPM could help increase the awareness of the surgical team leading to adaptation of surgical strategy or intraoperative assessment of the resection margin. The most frequently reported risk factors for a positive resection margin are shown in (Table 2). Large diameter of the tumour, advanced T classification, diffuse/mixed Lauren subtype and tumour location at the OGJ all increase the risk of a positive resection margin. In addition, Bissolati et al. found that a small macroscopic surgical margin of less than 2 cm for T1 and less than 3 cm for T2–T4 intestinal type tumours was an independent risk factor for a positive resection margin. This concurs with the recommendations of the Japanese guideline on gastrectomy for cancer [14, 15]. Van der Werf et al. also described a hospital volume of less than 20 gastrectomies per year as an independent risk factor. The benefit in high volume centres is likely to be multi-factorial encompassing staging accuracy, decision making and surgical experience [28].

**Table 2:** Risk factors for a positive resection margin in gastric and OGJ cancer

References	Tumour size	cT	OGJ location	Diffuse/mixed pattern	Signet ring cell	Grading	pT	pN
Cho et al. [13]	–	–	Yes	–	Yes	G3-G4	pT3-T4	pN1-N3
Lee et al. [25]	> 5 cm	–	Yes	–	Yes	–	–	–
Kim et al. [26]	–	–	Yes	Yes	–	–	–	–
Squires et al. [27]	> 6.9 cm	–	Yes	–	Yes	–	–	–
Bissolati et al. [14]	> 4 cm in T2-T4 diffuse/mixed pattern	–	Yes	Yes	Yes	–	pT3-T4	–
Van der Werf et al. [28]	–	cT3-T4	Yes	Yes	–	–	–	pN +
Kumazu et al. [29]	≥ 8 cm	–	Yes	Yes	–	–	pT4	–

## Does a positive proximal margin matter?

A systematic review and meta-analysis including 19,992 patients found an association of R1 status (circumferential, proximal or distal margin) with poorer overall survival (OS). A positive resection margin was also an independent prognostic factor irrespective of tumour stage [30]. Muneoka et al. analysed 2121 patients and reported a significantly worse 5 year OS and relapse-free survival (RFS) in patients with a positive resection margin in pT2-4 tumours, even when a re-resection was performed to obtain a definite R0 [31]. A worse 5 year recurrence-free survival (RFS) was not observed in patients with pT1 tumours, and only in these early tumours additive surgical resection was of benefit. This study and others [32, 33] found that a positive resection margin is associated with more advanced and aggressive tumours, which may explain the worse 5 year RFS. It would also explain why the recurrence pattern in patients with a positive resection margin is more frequently distant and not locoregional [31, 34]. Hence, worse OS and RFS in PPM cases would be related to an already advanced and aggressive disease, which relapses at distant sites earlier than from residual tumour at the resection margin. According to this theory, resection margin status would only impact on survival in early stage cancers, in which further treatments like re-resection may be of benefit.

## How to prevent a positive proximal margin?

Accurate preoperative assessment of the tumour is crucial to limit the chance of an incomplete resection. Endoscopy and computed tomography (CT), often combined with EUS, are mandatory for assessment of *T* status and the extent of infiltration into the oesophagus. Tumour-positive regional lymph nodes in the mediastinum may also guide the surgeon in defining the surgical approach and as a result the oesophageal resection margin [35]. The value of PET/CT scan for clinical staging of OGJ cancer is not clear but may be used to exclude distant metastases and to evaluate response after neoadjuvant therapy [36]. One of the issues is that signet ring cancers tend to be less FDG avid and so the sensitivity for PET in these high risk tumours (even for longitudinal extension) may be reduced [37].

Data about the biological nature of the tumour in addition to tumour location may be helpful, although there are no clear guidelines yet. Poorly differentiated tumours and the presence of signet ring cells should raise the suspicion of submucosal extension. Neoadjuvant therapy is indicated for locally advanced tumours. Both chemotherapy (FLOT) [38] and chemoradiotherapy (CROSS) [39] have shown improvements in locoregional control and a reduction of positive resection margin rates in OGJ cancers. Hence, neoadjuvant treatment may prevent a PPM in cardia cancer courtesy of tumour down-staging. However, surgical decision making with regard to longitudinal margins should still be guided by initial staging rather than relying on a down-staging effect. This would mandate an accurate assessment of tumour extent at the time of diagnosis.

There is no consensus about the length of macroscopic appearing normal oesophagus that should be resected [40]. Some studies suggested minimum of 2 cm [14, 41]. However, it can be challenging to resect more oesophagus via the abdomen and still leave room for a safe oesophagojejunostomy. Extension of the resection to a partial oesophagectomy or oesophagogastrectomy via a transthoracic approach (right thoracoscopy/thoracotomy or left thoracophrenolaparotomy––Sweet approach) might be needed. For such extended resections, the surgical team must have expertise.

Intraoperatively, manual palpation is helpful in open surgery but is not possible during minimally invasive procedures. Hence, the threshold for conversion (or alternative solutions) should be low if the complete resection cannot be assured by laparoscopy. Intraoperative endoscopy may help assess the proximal margin of the tumour. This could be used in minimally invasive and open approaches. Kawakatsu et al. described the systematic combination of preoperative placement of marking clips and intraoperative endoscopy to determine the surgical margin in patients who undergo laparoscopic extended gastrectomy. They achieved a success rate of 98.9% negative margins during the initial transection [42].

Intraoperative evaluation of the proximal resection margin by performing a frozen section analysis may be the best technique to assess margin status. The accuracy lies between 93 and 99% [27, 43, 44]. False negative results do occur especially in patients with submucosal spread of the tumour, poor differentiation (diffuse or mixed tumours), preoperative neoadjuvant therapy or surgical trauma due to the stapler device. However, intraoperative frozen section analysis is a time and resource-consuming technique, and a pathologist needs to be available in real time. According to current evidence [14, 36], frozen section analysis should be used selectively in patients where the risk of infiltrated margins is high and there is the opportunity to re-resect the oesophagus. This recommendation is in line with Japanese, Italian and AUGIS gastric cancer guidelines [15, 18, 22], in which frozen section examination is considered preferable to ensure an R0 resection in OGJ cancer. Risk factors (Table 2) could help decide in which patients frozen section analysis should be performed. Kumazu et al. defined 6 risk factors for a positive resection margin: remnant gastric cancer, oesophageal invasion, tumour size, undifferentiated type, macroscopic type 4 and pT4 disease. They observed a positive resection margin in 21.3% of their patients when four risk factors were present and 85.7% when five factors were identified. This led to their recommendation to perform a frozen section analysis in patients with four or more risk factors [29].

## How to manage a positive proximal margin?

When a positive proximal margin is diagnosed intraoperatively (e.g., by frozen section analysis), re-resection should follow. This may entail the need for changing the surgical approach as discussed earlier. When the proximal margin is positive at histopathological examination of the resection specimen after the operation, further treatments should be considered and tailored to tumour and patient characteristics. There is controversy regarding the benefit of performing a re-resection by a second operation. Some studies have described a lower incidence of local relapse after re-resection, but similar poor survival compared to patients that did not undergo a re-resection [27, 31]. According to Morgagni et al. a positive resection margin reflects advanced disease: regional and distant metastases are influenced by margin status and haematogenous and peritoneal metastatic relapse occurred earlier than local recurrence in patients with a positive resection margin [40]. Hence, some recommend re-resection when feasible and only for patients with N0 status or limited nodal involvement (less than 3–5 positive nodes) [13, 40, 45–47]. Furthermore, Bickenbach et al. suggested that only tumours with a limited depth of infiltration (≤ pT1–T2) may gain a survival benefit by re-resection [48].

Multimodal treatment with adjuvant chemoradiotherapy (CRT) has also been proposed as alternative to surgery. Stiekema et al. showed in a Dutch retrospective cohort study including 409 patients with a R1 resection (proximal, distal or circumferential) that adjuvant CRT was associated with improved survival when compared to patients without adjuvant CRT treatment [49]. In another study, the same authors analysed recurrence-free survival in gastric cancer patients treated with CRT after surgery concluding that an R1 resection was not an adverse prognostic factor [50]. A limitation of both studies was the low lymph node yield in most patients (< 15 nodes) and the heterogeneous population included. The survival benefit in the adjuvant CRT group could be due to better locoregional control in patients without an adequate (D2) lymphadenectomy. In addition, the finding that R1 status was not associated with RFS indicates that other more important factors dictate prognosis. Dikken et al. found that postoperative CRT had a major impact on local recurrence in resectable gastric cancer when a D1 lymphadenectomy was performed [51]. On the contrary, in patients with a D2 lymph node resection, adjuvant CRT did not shown any difference in local recurrence when compared to patients treated with surgery alone. Moreover, Ma et al. found that most patients with positive resection margin had advanced pathologic stage and that adjuvant treatment (28% CRT, 20% chemotherapy alone, 3% radiation alone, 1% reoperation) did not improve RFS or OS. The main failure pattern they found was distant recurrence (72%), suggesting that if patients are considered for adjuvant radiotherapy, they should be carefully selected [34]. Zhou et al. concluded that adjuvant CRT improves locoregional control and that nodal status may be the most important predictor for patient selection: only patients with pN0-2 disease may benefit from additional radiotherapy after R1 resection [52].

## Conclusion

Total gastrectomy for cancer aims to completely remove the primary tumour including locoregional lymph nodes. This is defined as achieving negative resection margins and a D2 nodal dissection. The best surgical strategy can only be planned after a comprehensive preoperative assessment of the patient (frailty, comorbidities, patient’s wishes) and location, stage and biological behaviour of the tumour. The surgeon should be involved in every step of this process. Awareness of risk factors for a positive resection margin could help in clinical decision making but unexpected intraoperative findings may lead to adaptation of the surgical management. When a positive resection margin is involved, further treatments options, such as re-resection and/or (chemo) radiation, should be carefully considered in selected cases.

## Data Availability

All data generated or analysed during this study are included in this published article.
